# The Kappa Opioid Receptor: From Addiction to Depression, and Back

**DOI:** 10.3389/fpsyt.2014.00170

**Published:** 2014-12-08

**Authors:** Laurence Lalanne, Gulebru Ayranci, Brigitte L. Kieffer, Pierre-Eric Lutz

**Affiliations:** ^1^CNRS UMR-7104, Translational Medicine and Neurogenetics, Institut de Génétique et de Biologie Moléculaire et Cellulaire, INSERM U-964, Université de Strasbourg, Illkirch, France; ^2^Department of Psychiatry, University Hospital of Strasbourg and Medical School of Strasbourg, Strasbourg, France; ^3^Douglas Mental Health Institute, McGill University, Montréal, QC, Canada

**Keywords:** kappa opioid receptor, place conditioning, reward, addiction, anhedonia, depression, comorbidity, animal models

## Abstract

Comorbidity is a major issue in psychiatry that notably associates with more severe symptoms, longer illness duration, and higher service utilization. Therefore, identifying key clusters of comorbidity and exploring the underlying pathophysiological mechanisms represent important steps toward improving mental health care. In the present review, we focus on the frequent association between addiction and depression. In particular, we summarize the large body of evidence from preclinical models indicating that the kappa opioid receptor (KOR), a member of the opioid neuromodulatory system, represents a central player in the regulation of both reward and mood processes. Current data suggest that the KOR modulates overlapping neuronal networks linking brainstem monoaminergic nuclei with forebrain limbic structures. Rewarding properties of both drugs of abuse and natural stimuli, as well as the neurobiological effects of stressful experiences, strongly interact at the level of KOR signaling. In addiction models, activity of the KOR is potentiated by stressors and critically controls drug-seeking and relapse. In depression paradigms, KOR signaling is responsive to a variety of stressors, and mediates despair-like responses. Altogether, the KOR represents a prototypical substrate of comorbidity, whereby life experiences converge upon common brain mechanisms to trigger behavioral dysregulation and increased risk for distinct but interacting psychopathologies.

## Introduction

Addiction and depression are chronic relapsing disorders with devastating consequences for individuals and their social environment (1). Chronic exposure to drugs of abuse, as well as prolonged abstinence from these drugs, is strongly associated with lowered mood and a negative affective state. Conversely, in some individuals, depressed mood potently drives the consumption of euphoric psychoactive substances, a process referred to as self-medication. Accordingly, epidemiological studies have clearly demonstrated a marked comorbidity between addiction and depression (2, 3). This comorbidity is accompanied by greater functional disability, longer illness duration, less social competence, and higher service utilization. Therefore, understanding pathophysiological mechanisms underlying comorbidity has important therapeutic implications.

The present review will discuss numerous lines of evidence that have accumulated to document the kappa opioid receptor (KOR) as an important substrate in comorbidity between addictive and depressive disorders. The KOR belongs to the opioid system, a neuromodulatory system that is widely expressed throughout the central and peripheral nervous systems. The opioid system is composed of three G protein-coupled opioid receptors: mu (MOR), delta (DOR), and kappa (KOR), which under physiological conditions are activated by a family of endogenous peptides to inhibit neuronal activity. Among opioid peptides, dynorphins (encoded by the *Pdyn* gene) primarily activate the KOR and have very low affinity for MOR or DOR. Conversely, the other opioid peptides (endorphin and enkephalins) poorly interact with the KOR. Therefore, the dynorphin/KOR signaling pathway forms a distinct process within the opioid system (4, 5).

Opioid receptors tightly regulate motivational processes, and are identified as important players in psychiatric disorders characterized by reward dysfunction, such as addiction and depression (6, 7). Several exhaustive reviews have recently summarized data on the role of MOR and DOR in these disorders, and will be briefly mentioned when appropriate (6, 8–11). Our goal is to provide the reader with a historical and neuro-anatomical perspective on where, when, and how KORs are recruited in rodent models of addiction and stress-related psychopathology (12–16). First, we will summarize how the KOR progressively emerged as an anti-reward system that encodes dysphoria and limits motivational properties of drugs of abuse. Secondly, we will show that the KOR is recruited and activated during stressful experiences, thereby contributing to the emergence of depressive states (10, 17, 18). Finally, we will discuss two main aspects of how these roles of KOR in addiction, stress-related behaviors and depression have important implications for the understanding of comorbidity. On one hand, we will show that stress-induced recruitment of KOR signaling is a potent trigger of drug-seeking and relapsing behaviors. On the other hand, we will summarize data on KOR function in the particular context of depressive-like behaviors that emerge during chronic exposure to drugs of abuse, as well as during drug abstinence (19–21).

As a pathophysiological substrate of comorbidity, the KOR represents a promising pharmacological target (10, 18). Clinical trials are currently on-going to assess KOR antagonists as a treatment for depression (22), in particular in the context of addicted patients suffering from comorbid depressive conditions (23, 24). Building on rodent studies, we will discuss the potential of therapeutic strategies targeting the KOR.

## Kappa Opioid Receptor: An Anti-Reward, Dysphoric System

Interest in KOR pharmacology historically stemmed from the hope of developing analgesic compounds devoid of the classical abuse potential of MOR agonists, such as morphine. Unfortunately, early human studies exploring properties of KOR agonists reported potent dysphoric and psychomimetic effects (25, 26). While these results clearly decreased the therapeutic potential of KOR in the treatment of pain, they also urged preclinical researchers to explore these intriguing dysphoric effects.

Activation of the MOR is known to induce euphoria in human, and to produce reinforcement in animal models. Therefore, researchers hypothesized that MOR and KOR may have opposite effects in the regulation of motivational processes, potentially through the modulation of common neuronal pathways. This framework was initially explored using conditioned place preference (CPP) or conditioned place avoidance (CPA). In this Pavlovian conditioning paradigm, a drug is repeatedly paired with a set of environmental stimuli that progressively acquire positive (CPP) or negative (CPA) motivational properties. Following repeated conditioning sessions, the animal subsequently exhibits preference or avoidance on re-exposure to the environmental stimuli (in the absence of the drug), a behavior that depends on learning, motivational, and hedonic mechanisms. The seminal rat study by Shippenberg and Hertz (27) reported that, as hypothesized, systemic administration of the KOR agonist U69593 or morphine yielded opposite effects, respectively producing CPA and CPP. While morphine-induced CPP reflects its reinforcing properties, KOR-induced CPA suggested that this receptor might be an anti-reward mechanism that contributes to a bi-directional regulation of motivation and hedonic tone.

**Figure 1:**
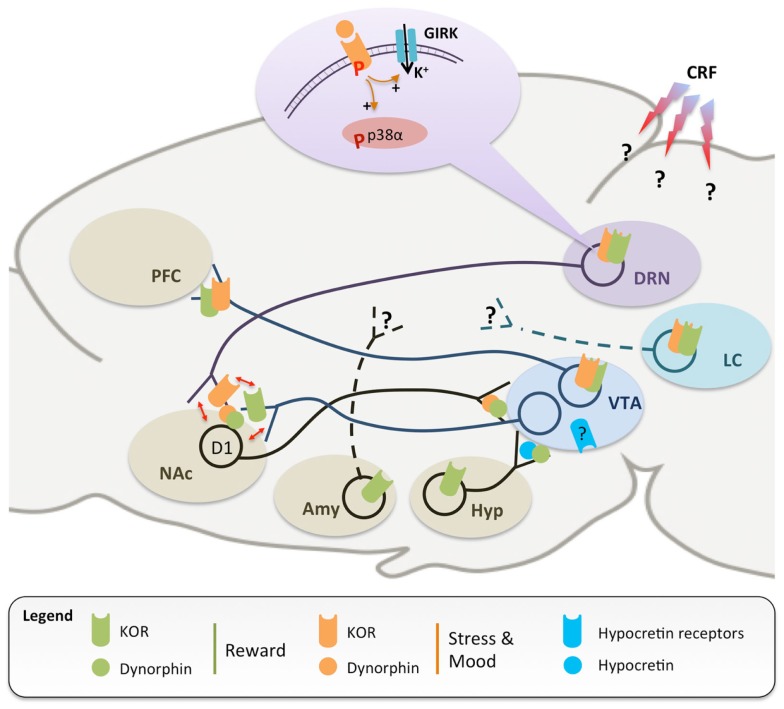
**A simplified scheme of neuronal circuits implicated in the regulation of reward (green) and stress (orange), which are both modulated by dynorphins and the kappa opioid receptor (KOR)**. KOR-mediated inhibition of ventral tegmental area (VTA) dopaminergic neurons projecting to the prefrontal cortex (PFC) is responsible for dysphoria and conditioned place aversion (13, 27, 33). Dynorphinergic medium spiny neurons, located in the nucleus accumbens (NAc) and expressing D1 dopamine receptors, send axonal projections back to the VTA (36), further supporting the importance of KOR in dopamine modulation and as an anti-reward agent. In addition, stressful experiences trigger widespread corticotropin releasing factor (CRF) release in the central nervous system (37), leading to dynorphin release and KOR phosphorylation, notably in the dorsal raphe nucleus (DRN) (38) and locus coeruleus (LC) (39). Stress-induced signaling events have been extensively characterized in the DRN, where activation of KOR stimulates G protein-coupled inwardly rectifying potassium channels [GIRK, see in Ref. (40)] and phosphorylation of the p38α kinase, in turn leading to translocation of the serotonin reuptake transporter to the plasma membrane and increased 5-HT reuptake (14). Similar stress-induced activation of KOR has also been documented at the level of the NAc, which appears to be the site where SERT translocation occurs (16). Available evidence also suggests that KOR regulation of 5-HT and DA neurotransmissions converge at the level of the NAc (red arrows), with important implications for comorbidity (see text for details). Further, recruitment of KOR signaling during stressful experiences has been shown: (i) in the amygdala, to potentiate conditioned place preference for drugs of abuse (20), and (ii) in the DRN (14) and LC (39), to mediate reinstatement of drug-seeking. KOR-dependent modulation of monoaminergic pathways has important implications for mood regulation. Systemic treatments with KOR agonist and antagonist have pro- and antidepressant-like effects, respectively. KOR activation locally in the NAc is sufficient to achieve a prodepressant-like effect (41–45), while knock-down of dynorphins in the NAc has opposite effect (46). Recently, hypocretin (blue) and dynorphin/KOR systems in the hypothalamus (Hyp) have been shown to stimulate and inhibit VTA DA neurons, respectively (47, 48). Avenues for future investigations include the identification of: (i) the signaling events following KOR activation in the LC and VTA; (ii) the brain regions receiving innervation from amygdala (Amy) and LC KOR-positive neurons; (iii) the brain sites where CRF acts to stimulate dynorphinergic neurons, and (iv) the neurochemical identity and projections targets of VTA neurons expressing hypocretin receptors. Altogether, data indicate that the KOR inhibits the activity of all three monoaminergic centers at multiple sites, thereby critically controlling their interactions in rodent models of addiction, depression, and dual diagnosis.

The next step was to investigate underlying neurochemical substrates, with early studies exploring how the KOR may regulate the mesolimbic pathway (Figure 1). This pathway is composed of dopaminergic (DA) neurons that are located in the midbrain ventral tegmental area (VTA) and project to forebrain limbic structures, including the ventral striatum [or nucleus accumbens (NAc)] and prefrontal cortex (PFC). Animal and human data have clearly demonstrated that drug addiction (and mood disorders, see Part 2) associate with major disruptions of the brain’s DA reward circuitry (28), which normally acts to predict and encode the salience of environmental stimuli and natural rewards. The now classical “unitary” theory of addiction postulates that essentially all drugs of abuse enhance DA transmission in the NAc, an effect that is central to their rewarding properties (29). Within this line, many studies have consistently shown that acute reinforcing effects of morphine rely on disinhibition, i.e., the activation of DA neurons. This disinhibition occurs through the activation of MOR expressed by GABAergic interneurons located mainly in the tail of the VTA [tVTA, or RMTg, see in Ref. (30, 31)], but also in the VTA and NAc (32). In contrast, decreased DA signaling was hypothesized to be responsible for the encoding of KOR-mediated aversion. Using microdialysis, Spanagel and colleagues (33) showed that DA release in the NAc was decreased by infusion of a KOR agonist into the NAc, but not into the VTA (pharmacological agents used in every study discussed in the present review are summarized in Tables 1–3). In addition, infusion of a KOR antagonist (nor-BNI) into the NAc increased DA release, suggesting that dynorphins tonically reduce DA neurotransmission in this region. The most compelling evidence implicating DA neurons in KOR-induced aversion came recently (12) from the use of genetically modified mice using the Cre-lox recombination system (34). Bals-Kubik et al. took advantage of a knockin mouse expressing the Cre-recombinase under transcriptional control of the endogenous promoter of the DA transporter [DAT, a specific marker of DA neurons (35)]. These mice were bred with another knockin mouse harboring a conditional “floxed” KOR allele, thereby achieving the specific deletion of KOR in DA neurons (DAT KOR-cKO). At the behavioral level, KOR-induced CPA was abolished in DAT KOR-cKO mice, and restored upon virally mediated KOR re-expression in the VTA (12).

**Table 1:** **Kappa opioid receptor function in reward regulation**.

Species/model	KOR-targeting compound	Behavioral paradigm	Route of administration (dose)	Timing of administration	Outcome of behavioral paradigm	Reference
Rats	U69593	CPP + CPA	s.c. (0.16 mg/kg)	Prior to drug conditioning	KOR-induced aversion	(27)
Rats	U50488H	CPA	Intra-NAc (10 μg)	Prior to drug conditioning	KOR-induced aversion	(49)
			Intra-VTA (0.33, 1 μg)			
			Intra-PFC (1, 3.3 μg)			
			Intra-LH (3.3 μg)			
Rats	U50488H	Social play	i.p. (1, 3 mg/kg)	1 h prior to testing	Reduced social play	(74)
Rats	U50488H	Cocaine SA	i.p. (1.2, 2.5 mg/kg)	5 min prior to testing	Dose-dependent decrease in SA	(60)
Mice	U50488H	Morphine SA	i.p. (5, 10 mg/kg)	15 min prior to testing		
Rhesus monkeys	EKC	Cocaine SA	i.v. (0.01, 0.032 mg/kg/h)	10 consecutive days (23 h/day)	Dose-dependent decrease in SA	(65)
	U50488H		i.v. (0.1 mg/kg/h)		Reduced SA	
Rats	U50488H	EtOH oral SA (24 h access)	i.p. (10 mg/kg)	6 h prior to drinking measures	Reduced EtOH drinking	(62)
Rhesus monkeys	Enadoline	Cocaine SA	i.v. (0.001, 0.0032 mg/kg/h)	10 consecutive days (23 h/day)	Dose-dependent decrease in SA	(66)
	Mr2033		i.v. (0.0032, 0.01 mg/kg/h)			
	Bremazocine		i.v. (0.0032 mg/kg/h)		Reduced SA	
Rats	U69593	Cocaine SA	s.c. (0.32 mg/kg)	15 min prior to testing	Reduced SA	(68)
Rats	U69593	Cocaine-reinforced cocaine SA	s.c. (0.32 mg/kg)	15 min prior to testing	Reduced maintenance of SA	(67)
Rats	U50488H	EtOH SA (2 h access)	i.p. (2.5, 5, 10 mg/kg)	15 prior to ethanol access	Dose-dependent decrease in EtOH intake	(63)
Rats	U69593	ICSS	i.p. (0.25, 0.5 mg/kg)	45 min prior to testing	Reduced ICSS threshold	(78)
Mice	Salvinorin A	CPA	i.p. (1, 3.2 mg/kg)	Prior to drug conditioning	KOR-induced aversion	(85)
Mice	nor-BNI	WIN 55.212-2 SA	s.c. (5 mg/kg)	4 hr prior to first session	Enhanced maintenance of self-administration	(70)
Pdyn KO mice	Ø	WIN 55.212-2 SA	Ø	Ø		
Pdyn KO mice	Ø	Nicotine SA	Ø	Ø	Enhanced acquisition of nicotine SA	(69)
Mice	U50488H	EtOH CPP	i.p. (1, 3 mg/kg)	10 min prior to drug conditioning	Reduced EtOH CPP	(64)
Rats	Salvinorin A	ICSS	i.p. (2 mg/kg)	45 min prior to testing	Increased ICSS threshold with lowered breakpoint	(83)
Pdyn KO mice	Ø	EtOH CPP	Ø	Ø	Increased EtOH CPP	(72)
	Ø	EtOH SA (24 hr access)	Ø	Ø	Increased EtOH drinking	
DAT KOR-cKO mice	U69593	CPA	s.c. (0.32 mg/kg)	Prior to drug conditioning	No KOR-induced aversion	(12)
Rats	U50488	ICSS	i.p. (5, 10 mg/kg)	45 prior to testing	Higher dose-dependent increase in ICSS threshold in female than male rats	(87)
Mice	U50488	CPA	i.p. (2.5, 10 mg/kg)	Prior to each drug conditioning session	Dose-dependent difference in aversion between females and males	(76)

*This table lists papers discussed in Part 1 of the main text. CPA, conditioned place aversion; CPP, conditioned place preference; SA, self-administration; ICSS, intracranial self-stimulation; EtOH, ethanol; LH, lateral hypothalamus; NAc, nucleus accumbens; PFC, prefrontal cortex; VTA, ventral tegmental area; nor-BNI, norbinaltorphimine; EKC, ethylketocyclazocine*.

**Table 2:** **Kappa opioid receptor function in mood regulation**.

Species/model	KOR-targeting compound	Behavioral paradigm	Route of administration (dose)	Timing of administration	Outcome of behavioral paradigm	Reference
Rats	nor-BNI	LH	Local into the NAc (1 μg/side)	72 h prior to testing	Reduced failure number and latency to escape	(88)
Rats	nor-BNI	FS	i.c.v. (20 μg)	72 h prior to testing	Reduced immobility and increased swimming	(42)
	GNTI					
Rats	ANTI		i.p. (0.3–3 mg/kg)	23 h prior to testing	Reduced immobility	
Pdyn KO mice	Ø	FS-induced analgesia	Ø	Ø	Loss of FS-induced analgesia	(90)
Mice	nor-BNI		i.p. (10 mg/kg)	60 min prior to FSS		
Rats	JDTic	FS	s.c. (0.3–3 mg/kg)	23 h prior to testing	Reduced immobility with increased swimming	(41)
	nor-BNI		s.c. (1, 10 mg/kg)			
Pdyn KO mice	Ø	SD-induced analgesia		Ø	Loss of SD-induced analgesia	(92)
Mice	nor-BNI		i.p. (10 mg/kg, daily)	1 h prior to first SD trial		
Rats	nor-BNI	FS	i.c.v. (20 μg/kg)	24 h prior to testing	Reduced immobility with increased swimming	(99)
Pdyn KO mice	Ø	FS-paired odorant aversion & Footshock CPA	Ø	24 h prior to testing	No aversion from FS-paired odor or footshock	(37)
Mice	nor-BNI		i.p. (10 mg/kg)	
Mice	nor-BNI	SD-induced analgesia	Local into the DRN (2.5 μg/side)	5–7 days prior to testing	Loss of SD-induced analgesia	(38)
Mice	Buprenorphine	FS	i.p. (0.25 mg/kg)	24 h prior to testing	Reduced immobility	(45)
		Novelty-induced hypophagia			Decrease in latency to approach	

*This table lists papers discussed in Part 2 of the main text. CPA, conditioned place aversion; LH, learned helplessness; FS, forced swim; SD, social defeat; NAc, nucleus accumbens; DRN, dorsal raphe nucleus; nor-BNI, norbinaltorphimine; GNTI, 5′-guanidinonaltrindole; ANTI, 5-acetamidinoethylnaltrindole*.

**Table 3:** **Kappa opioid receptor function at the interface of reward and mood regulation**.

Species/model	KOR-targeting compound	Behavioral paradigm	Route of administration (dose)	Timing of administration	Outcome of behavioral paradigm	Reference
Mice	nor-BNI	FS-induced cocaine CPP	i.p. (10 mg/kg)	1 h prior to testing	Loss off FS-induced potentiated preference	(90)
Pdyn KO mice			Ø	Ø		
Rats	JDTic	Footshock-induced/cocaine-primed reinstatement of cocaine SA	i.p. (10, 30 mg/kg)	24 h prior to testing	Loss of footshock-induced reinstatement	(41)
Mice	U50488H	Cocaine CPP	i.p. (5 mg/kg)	360, 60, 30, 15 min prior to testing	Time-dependent potentiation or suppression of preference	(105)
KOR KO mice	Ø	FS-induced cocaine CPP	Ø	Ø	Loss off FS-induced potentiated preference	
Mice	nor-BNI	SD-induced cocaine CPP	i.p. (10 mg/kg)	1 h prior to testing	Loss off SD-induced potentiated preference	(92)
Pdyn KO m*ce*	Ø		Ø	Ø		
Mice	nor-BNI	Footshock-/FS-induced/cocaine-primed reinstatement of cocaine CPP	i.p. (10 mg/kg)	1 h prior to testing	Loss off footshock- and FS-induced reinstatement	(104)
Pdyn KO mice	Ø		Ø	Ø	
KOR KO mice						
Mice	nor-BNI	SD-induced reinstatement of cocaine CPP	Local into the DRN (2.5 μg/side)	5–7 days prior to testing	Loss of SD-induced reinstatement	(38)
Mice	Zyklophin	FS-induced/cocaine-primed reinstatement of cocaine CPP	s.c. (3 mg/kg, daily)	60 min prior to FSS exposure	Loss off FS-induced reinstatement	(103)
Mice	nor-BNI	FS-induced reinstatement of EtOH SA	i.p. (10 mg/kg)	60 min prior to FSS exposure	Loss of FS-induced self-administration	(106)
Pdyn KO mice	Ø		Ø	Ø		
Rats	nor-BNI	EPM after 24 h of withdrawal from EtOH	i.p. (20 mg/kg)	24 h prior to testing	No reduced open arm exploration	(125)
	U50488	EPM	i.p. (10 mg/kg)	10 min prior to testing	Reduced open arm exploration	
Rats	nor-BNI	FS-induced reinstatement of cocaine SA	Local into the VTA (2.5 μg/side)	24 h prior to FSS exposure	Loss of FS-induced reinstatement of self-administration	(112)
Rats	nor-BNI	EPM after 6 weeks of withdrawal from EtOH	i.p. (20 mg/kg)	24 h prior to testing	No reduced open arm exploration	(135)
	U50488		i.p. (10 mg/kg)		Reduced open arm exploration	
Mice	nor-BNI	U50488-induced reinstatement of cocaine CPP	Local into the LC (2.5 μg/side)	5–7 days prior to testing	Reduced KOR-induced reinstatement	(39)
Mice	U50488	FS-induced reinstatement of cocaine CPP	i.p. (5 mg/kg)	30 min prior to testing	Enhanced FS-induced reinstatement	(162)
Rats	nor-BNI	Yohimbine-induced reinstatement of heroin SA	i.p. (20 mg/kg)	48 h prior to testing	Reduced yohimbine-induced reinstatement	(109)
Rats	nor-BNI	FS-induced reinstatement of cocaine SA	i.p. (10 mg/kg)	22 h prior to FSS exposure	Loss of FS-induced reinstatement of self-administration	(113)
KOR KO mice	Ø	Social behaviors after 4 weeks of withdrawal from heroin	Ø	Ø	Loss of social avoidance	(139)
Mice	LY2456302	FS	*per os* (10 mg/kg)	60 min prior to testing	Reduced immobility	(144)
Rats		EtOH SA	*per os* (3, 10 mg/kg, daily)	60 min prior to testing	Dose-dependent decrease in EtOH consumption	

*This table lists papers discussed in Part 3 of the main text. FS, forced swim; CPP, conditioned place preference; SA, self-administration; SD, social defeat, EPM, elevated plus maze; EtOH, ethanol; DRN, dorsal raphe nucleus; VTA, ventral tegmental area; LC, locus coeruleus; nor-BNI, norbinaltorphimine*.

In parallel, investigators undertook a brain-wide analysis of regions where recruitment of the KOR may potentially encode aversion. The effect of local KOR activation was assessed in several areas using the CPA paradigm (49). Infusion of the KOR agonist U50,488H in the NAc was sufficient to induce a robust CPA, consistent with the notion that KOR activation in this region decreases DA release. Surprisingly, infusions in the PFC, lateral hypothalamus, and VTA (but not in the substantia nigra and dorsal striatum) had similar effects, suggesting that multiple KOR pools may regulate motivation and hedonic tone. These results also indicate that activation of VTA KOR induces CPA in the absence of any change in NAc DA release [see aforementioned neurochemical data (33)], implying the involvement of another brain region receiving DA innervation (i.e., the PFC, see below). In addition to the regulation of DA transmission, KOR expression and function is now under investigation in many other brain regions using rodent assays relevant to reward and mood [e.g., bed nucleus of the stria terminalis, BNST, amygdala, locus coeruleus (LC), see below].

An important next goal was to identify which neuronal cell types are controlled by the KOR. Electron microscopy approaches (50, 51) found that in the NAc, half of the axons that were KOR-immunoreactive also expressed DAT. Interestingly, this study found that almost one-third (29%) of these KOR-immunoreactive axons were DAT-negative but contacted pre-synaptic terminals of DAT-positive neurons, suggesting that the mesolimbic pathway is regulated at the level of the NAc by afferent neurons expressing the KOR. Based on recent evidence (16), it is likely that non-DAergic KOR-positive neurons are, at least in part, serotonergic (5-HT). Possibly, KORs expressed by 5-HT neurons may mediate DA/5-HT crosstalk in the NAc, and represent a mechanism that contributes to interactions between mood and reward, as well as between addiction and depression (see below). At the level of the NAc (52), there is also evidence for KOR-dependent modulation of glutamate release, suggesting that this receptor may be expressed pre-synaptically by glutamatergic cortical neurons that densely innervate the NAc. To our knowledge, the behavioral relevance of the latter KOR pool has not been addressed. Finally, in the PFC (53), the KOR was mainly located on pre-synaptic terminals, likely corresponding to DAergic inputs, although the neurochemical identity of these neurons was not assessed.

Other investigators used electrophysiology and immunohistochemistry to identify KOR-expressing neurons. Application of a KOR-selective agonist in the VTA decreased spontaneous firing activity of a sub-group of neurons (54). This KOR-mediated inhibition only occurred in DA cells, as indicated by immunoreactivity for tyrosine hydroxylase (the rate limiting enzyme for DA synthesis, and another marker of DA neurons). Electrophysiology, retrograde tracing and microdialysis were then combined to assess whether DA neurons projecting either to the NAc or to the PFC are differentially regulated by the KOR (55). These elegant experiments revealed that local KOR activation in the VTA hyperpolarized PFC-targeting DA neurons, but had no effect on NAc-targeting DA neurons. Accordingly, DA release was reduced in the PFC, but not in the NAc, upon VTA KOR activation.

Overall these results are consistent with previous CPA and microdialysis studies, and suggest a model whereby VTA KORs do not control NAc DA tone but rather modulate DA release in the PFC to produce CPA. The previously described DAT KOR-cKO mice recently provided strong evidence for the latter hypothesis. Tejeda et al. found that infusion of a KOR agonist in the PFC decreased DA overflow in wildtype (WT) but not in DAT KOR-cKO mice (13), confirming KOR-mediated control of DA transmission in the PFC. Importantly, the authors then directly tested the behavioral relevance of PFC KORs for dysphoria in rats. Infusion of a KOR antagonist into the PFC was sufficient to prevent KOR agonist-induced CPA, clearly identifying the limbic cortex as a necessary substrate for this behavioral effect.

Collectively, results from these various methodological approaches also suggest that NAc-projecting DA neurons express KOR in pre-synaptic terminals, but not in soma and dendrites (33), while PFC-projecting DA neurons express KOR in both compartments (13, 54, 55) (Figure 1). At the molecular level, it is currently unknown how DA neurons may control KOR trafficking to distinct cellular compartments as a function of their projection targets. We speculate that the type of electrophysiological feedback (excitatory from the cortex, inhibitory from striatal medium spiny neurons) provided to the VTA by each region may be implicated. Alternatively, cell-autonomous processes might be involved, with distinct transcriptomic profiles in NAc- and PFC-projecting DA neurons leading to distinct KOR post-translational modifications and trafficking. To experimentally address the latter hypothesis, technological advances now allow researchers to distinguish transcriptomes from neuronal populations sharing a common cell-body location but with distinct projections (56). Alternatively, retrograde tracing may be coupled with knockin reporter mice expressing opioid receptors in fusion with fluorescent proteins [such mice are currently available for mu and delta, but not kappa, opioid receptors (57, 58)].

Activity of the dynorphin/KOR pathway on DA neurotransmission and in CPA has obvious implications for addiction-related behaviors, as observed for a variety of drugs of abuse in self-administration paradigms [see in Ref. (59) for an exhaustive review]. KOR agonists dose-dependently decrease morphine self-administration in rats and mice (60, 61). Similar inhibitory effects of KOR activation were found for ethanol (62–64), cocaine (61, 65–68), nicotine (69), and cannabis (70), and these were associated with reduced drug-induced DA release (for example, cocaine, see in Ref. (71); ethanol, see in Ref. (72)). Globally, these results provide robust evidence for an inhibitory effect of KOR on the rewarding effects of drugs of abuse, and recent findings suggest that natural rewards, such as social interactions, may also be affected. In prairie voles, a monogamous rodent species, maintenance of mating pair bonds relies on the expression of aggressive behaviors toward novel conspecifics. Interestingly, this form of “social aversion” has been shown to be mediated by KOR signaling within the NAc (73). In rodents, social play represents a highly studied, naturally occurring behavior that recruits DA neurons and triggers potent reinforcement. Systemic activation of KOR decreased social play in both rats (74, 75) and mice (76). These findings are relevant to our understanding of depression in human as anhedonia, or the altered perception of rewarding properties of everyday-life stimuli (including social interactions), is a hallmark of this condition. Therefore, while KOR-dependent modulation of DA and reward was initially conceptualized and explored in addiction paradigms, it is now becoming clear that it also has strong implications for mood disorders (17, 18). CPA reflects the interaction of several neurobiological mechanisms, corresponding to three psychological constructs: learning, motivation, and hedonia. Intra-cranial self-stimulation (ICSS) is another paradigm assessing these three aspects: in this operant conditioning, animals learn to self-administer brief electrical pulses into specific brain regions (usually the medial forebrain bundle (77)). Systemic activation of KOR was found to induce an anhedonic-like state in ICSS, as indicated by increased stimulation threshold (78). In the latter work, the stimulation electrode was placed in the lateral hypothalamus, strengthening previous evidence for KOR-dependent regulation of hedonic state occurring outside the NAc (49). Further, Potter et al studied the kinetics of KOR agonist-induced ICSS modulation following acute and repeated injections (79). The KOR agonist Salvinorin-A increased the stimulation threshold, and this acute effect persisted with daily injections over an 8-day period. Interestingly, repeated injections also triggered delayed and opposite effects, as evidenced by decreased ICSS stimulation threshold 24 h post-injection, suggesting that opponent processes (80, 81) had developed.

The neuronal pathway potentially linking hypothalamic KOR activity with DA transmission and reward has been poorly studied. Recent elegant data using electron microscopy, electrophysiology, ICSS, and cocaine self-administration (47, 48), suggest an antagonistic interplay between orexin and dynorphin peptidergic systems. The hypocretin/orexin system is composed of neurons originating in the lateral hypothalamus and projecting to several mesolimbic structures (82). Importantly, orexin and dynorphin were found to act as co-transmitters in neurons of the hypothalamus (47): the two peptides co-localize in synaptic vesicles, and are co-released upon electrical hypothalamic stimulation. The authors further showed that orexin and dynorphin act within the VTA to stimulate and inhibit, respectively, the excitability of DA neurons, thereby bi-directionally modulating reward (in ICSS experiments), and self-administration of cocaine (and potentially other drugs of abuse). In the VTA, most cells (65%) were found to be common targets for both orexin and dynorphin. Based on previous evidence, future experiments may test the hypothesis that VTA DA neurons expressing both KOR and orexin receptors project preferentially to the PFC rather than the NAc.

Overall, data on KOR function in the regulation of reward highlights the importance of assessing the full spectrum of peptides and neurotransmitters expressed along the mesolimbic pathway and associated neuronal circuits. Determining how this network dynamically evolves under chronic pathologic conditions will be an exciting endeavor.

## Kappa Opioid Receptor: A Stress System Implicated in Depression Pathophysiology

In parallel to these studies on reward, recent data have demonstrated that the KOR also controls emotional responses, in particular during stressful experiences. Pharmacological studies in rodents indicate that the dynorphin/KOR system regulates mood-related behaviors. In rats, systemic administration of KOR agonists and antagonists showed pro- and antidepressant-like effects, respectively, in the forced swim (FS) and learned helplessness (LH) tests [see in Ref. (10, 41–45) for a review]. Consistent with CPA studies, systemic KOR activation decreased DA release in ventral (44, 83), dorsal (84, 85), and striatal regions, while local NAc injection of a KOR agonist mimicked the prodepressant-like effect of systemic treatment (86). These data further confirm that KOR-dependent modulation of DA is implicated in both mood- and addiction-related behaviors (28). Interestingly, KOR-dependent prodepressant-like effects may be modulated by gender (87), an important aspect considering that the prevalence of depression is higher in women. Using ICSS, the authors found that the KOR agonist-induced increase in ICSS stimulation threshold was higher in male than female rats. This effect was independent from circulating levels of gonadal hormones, and was not accounted for by sex differences in pharmacokinetics of the agonist. Rather, sex differences in KOR agonist-induced neuronal activation, as revealed by c-fos staining, were found in the BNST and PVN, but not in the NAc or amygdala. Therefore, in addition to the mesolimbic pathway, sex-specific KOR-dependent regulation of hedonic tone may also occur at the level of the BNST and PVN, two structures controlling stress-responses and emotions.

Adding to pharmacological studies targeting KOR, there is also evidence that dynorphins provide an endogenous tonic regulation of mood-related traits (43, 88). In the NAc, medium spiny neurons expressing the DA D1-receptor are known to synthesize and release dynorphins under the control of the cAMP response element binding protein (CREB). Accordingly, prodynorphin levels were decreased in the NAc of transgenic mice overexpressing a dominant negative form of CREB. This effect was associated with decreased behavioral despair in the LH paradigm. Consistently, a recent study reported that *Pdyn* knock-down (by viral expression in the NAc of an anti-*Pdyn* short hairpin RNA) decreased depressive-like behavior in the FS test (46).

Beyond baseline emotional responses, data indicate that activity of the dynorphin/KOR system is potentiated by stress. Acute, but not chronic, restraint stress was shown to sensitize KOR-dependent CPA (89). Also, repeated exposure to FS stress produced a prodepressant-like effect that was blocked by the KOR antagonist nor-BNI, and was absent in *Pdyn* KO mice (90). Dynorphins were further demonstrated to modulate repeated stress-dependent aversive conditioning (37). Mice trained to associate a given odor with FS stress robustly avoided that odor. This avoidance behavior was not observed in *Pdyn* KO mice, and was blocked in WT mice by pre-treatment with a KOR antagonist. Similarly, a context repeatedly paired with footshocks was aversive in WT mice; but again, this effect was absent in *Pdyn* KO mice, and prevented by KOR antagonist pre-treatment. Importantly, the authors showed that corticotropin releasing factor (CRF) release in the central nervous system is likely the primary event responsible for stress-induced recruitment of the dynorphin/KOR system. Results indicated that systemic injection of CRF triggered KOR phosphorylation, as revealed using a phospho-KOR antibody. Further, stress-induced CPA (mimicked by systemic or intracerebroventricular injection of CRF or the CRF_2_-receptor agonist Urocortin III) was absent in *Pdyn* KO mice, and blocked by nor-BNI pre-treatment. Following stress exposure, KOR activation, and phosphorylation was identified in several brain structures, including the basolateral amygdala, hippocampus, dorsal raphe, VTA, and NAc. Altogether, these data show that dysphoric aspects of stress behaviorally manifest when CRF stimulates dynorphin release, yielding KOR activation (37).

Stress is a complex physiological process that has a primarily adaptive value, but that can trigger pathological events during prolonged and excessive stressful experiences. Recently, interactions between stress and the KOR have been investigated using more sophisticated and ethologically relevant models of depression. In nature, confrontation among conspecific animals potentially generates significant consequences in terms of control over resources, access to mates, and social positions. For example, the resident–intruder social defeat paradigm (91) is a naturalistic model characterized by potent aggressive interactions that are unpredictable and inescapable, thereby inducing several anhedonia-like symptoms such as diminished sexual pursuit and decreased sucrose preference (52). McLaughlin and colleagues were the first to reveal the role of dynorphins and KOR in transducing the effects of social stress (92). Mice exposed to repeated social defeats over 3 days showed a characteristic defeated postural response, as well as an increased nociceptive threshold, or stress-induced analgesia (SIA, observed in a tail withdrawal latency assay). Both aspects were prevented in mice pre-treated with a KOR antagonist, or lacking the *Pdyn* gene. Another important feature of the social defeat model is that its effects show high inter-individual variability, both in rats and among inbred mice, such that animals can be typically separated into susceptible and resilient groups (93). Along this line, according to Bérubé et al. (94), expression levels of dynorphins in the NAc differ among susceptible and resilient Sprague–Dawley rats. Increased dynorphin mRNA levels (measured by qPCR) were found in the ventral striatum of susceptible rats (NAc shell, coherent with previous mice data), while surprisingly increased levels were observed in the dorsal striatum of resilient individuals, suggesting that the regulation of DA and mood by dynorphin and KOR may be more complex than anticipated. Additional studies will be necessary to further substantiate this hypothesis. In contrast, another study reported no change in dynorphin levels in VTA or NAc of socially defeated Long–Evans rats (95). Discordance between these two studies might be explained by the different strains used, or the absence of a distinction between resilient and susceptible Long–Evans rats in the latter study. Of note, Nocjar et al. (95) found decreased dynorphin-A, as well as decreased orexins A and B, in the hypothalamus of defeated rats. Therefore, combined regulation of VTA DA neurons activity by these two antagonistic peptides might mediate defeat-induced KOR-dependent social aversion, and be impaired following social defeat.

We previously discussed (Part 1) how the KOR may display differential cellular localization across the two populations of VTA DA neurons projecting to the NAc or to the PFC. A recent report suggests that this anatomical dissociation may have relevance for the understanding of the effects of chronic social defeat. Chaudhury and colleagues (96) showed that the selective inhibition of VTA DA neurons projecting either to the NAc or to the PFC, respectively, promoted resilience or susceptibility to repeated social defeat. Due to its selective cellular localization, it is tempting to speculate that the KOR may mediate prodepressant-like symptoms induced by the inhibition of the VTA-PFC DA pathway.

In addition to DAergic signaling, new findings suggest that 5-HT transmission may also be modulated by KOR in stress- and social defeat-based models of depression. Electrophysiology experiments (97, 98) initially demonstrated that the KOR regulates 5-HT neurons at the level of the dorsal raphe nucleus (DRN), a main 5-HT brain nucleus. Importantly, rescue experiments showed that the selective re-expression of KOR in the DRN of KOR KO mice is sufficient to restore the CPA induced by infusion of a KOR agonist in the NAc (38). Together with previous findings, these results indicate that KOR in the PFC, and KOR expressed by neurons present in the DRN, which target the NAc (that are likely to be 5-HT neurons), are necessary and sufficient, respectively, for the expression of KOR agonist-induced aversion. At the molecular level, acute social defeat was shown to trigger phosphorylation of KOR and the p38α kinase in the DRN (14). Recruitment of p38α in 5-HT neurons is essential, as defeat-induced social avoidance was abolished in cKO mice in which p38α is specifically deleted from serotonin transporter (SERT)-expressing neurons (p38α-cKO^SERT^). Phosphorylated p38α in turn promotes SERT translocation to the plasma membrane, thereby increasing 5-HT reuptake and likely mediating social avoidance. Electrophysiological recordings in brain slices (40) also showed that KOR activation dampens excitability of DRN 5-HT neurons through two mechanisms: the pre-synaptic inhibition of glutamatergic inputs, and the post-synaptic stimulation of G-protein-gated inwardly rectifying potassium channels (GIRKs). Repeated exposure to FS stress impairs post-synaptic, but not pre-synaptic, effects of KOR activation. Importantly, stress-induced inhibition of KOR-mediated GIRK currents was abolished in p38α-cKO^SERT^ mice. Finally, recent evidence suggests that KOR regulation of DA and 5-HT neurons may converge at the level of the NAc to produce dysphoric and depressive-like effects. Repeated FS stress selectively increased cell-surface expression of SERT in the ventral striatum, but not in other regions examined (dorsal striatum, hippocampus, PFC, amygdala, or DRN). This effect of stress on SERT was prevented by pharmacological blockade of KOR signaling in the NAc, but not in the DRN (16). Altogether, stressful experiences appear to recruit a CRF-dynorhin-KOR-p38α-GIRK signaling cascade within DRN 5-HT neurons, as well as KOR activation in the NAc. These molecular adaptations in turn lead to up-regulation of SERT function in the NAc, and ultimately affect DA function to produce behavioral symptoms. Whether similar DRN signaling is also involved in more prolonged mood-related deficits, in particular in the context of chronic exposure to drugs of abuse (Part 3), has yet to be determined.

In addition to 5-HT and DA circuits, other possible sites of KOR-dependent mood regulation notably include hippocampal neurogenesis and noradrenergic (NA) transmission. One report found that in rats, the antidepressant-like effect of the KOR antagonist nor-BNI (99) associated in the hippocampus, as well as in other structures (e.g., frontal cortex, amygdala, hippocampus, and endopiriform cortex), with increased mRNA levels of BDNF, a neurotrophic factor controlling synaptic plasticity and neurogenesis. Further studies are required to better understand the relevance of this KOR/BDNF interaction.

## Kappa Opioid Receptor at the Interface of Depression and Addiction

We have summarized the role of KOR in the regulation of reward processes (Part 1), and in the modulation of stress-responses and affective states (Part 2). Based on these data, several groups have recently explored how the KOR may mediate the interplay between addiction and depression. The relationship between these two disorders is likely bi-directional: addicts show a strong lifetime risk for anxiety or depressive disorders, while, conversely, depressed patients frequently abuse drugs to self-medicate their depressive symptoms. Both aspects are currently being addressed in animal models.

### Stress sensitivity, relapse, and the emergence of depressive symptoms in addicted individuals

#### Stress-induced relapse during the course of addiction

Rodent models of CPP and drug self-administration have been extensively used to investigate various triggers for relapse, or the re-initiation of drug-seeking behaviors. Following a period of repeated conditionings, or stable drug self-administration, animals are repeatedly re-exposed to CPP or operant chambers in the absence of drug, so that drug-seeking and instrumental responding are no longer reinforced and progressively disappear, a process referred to as extinction. Importantly, after extinction has been achieved, relapse can be triggered through re-exposure (i.e., “priming”) to the drug of abuse (drug-induced reinstatement), or through exposure to an acute stressor (stress-induced reinstatement). Classically, stressful experiences represent major lifetime risk factors for the emergence of depressive (100) and addictive (101) disorders. In addition, drugs of abuse potentiate the neurobiological and behavioral effects of a variety of stressors, which in turn may potentiate the effects of drugs of abuse in a vicious circle (see below the stress-induced reinstatement of CPP) (102). Therefore, addiction and stress interact tightly, and the underlying neurobiological mechanisms represent factors contributing to the comorbidity between addiction and stress-related psychopathology.

Based on available evidence implicating KOR in stress effects (Part 2), researchers went on to probe the role of this receptor in stress-induced reinstatement. Overall, results demonstrate that KOR signaling critically mediates stress-induced reinstatement for a variety of drugs of abuse. In rats, pre-treatment with a KOR antagonist (either JDTic or nor-BNI) significantly decreased stress-induced (footshock), but not cocaine-induced, reinstatement of cocaine self-administration (41). In mice, similar findings were obtained for both stress- and drug-induced reinstatement of cocaine-seeking in a CPP assay [using a new systemically active KOR peptidergic antagonist with short duration of action (103)]. Further, exposure to acute or repeated stress reinstated cocaine CPP in WT, but not in KOR or *Pdyn* KO mice, nor following pharmacological KOR blockade (104). Stress and the KOR also interact at the level of cocaine-context associative conditionings: stress is classically known to potentiate cocaine CPP, and this effect is mimicked by systemic KOR activation (105). Therefore, the KOR mediates stress/cocaine interactions during initial drug exposure, as well as following extinction.

Consistent with cocaine data, genetic and pharmacological approaches showed that stress-induced reinstatement of ethanol consumption similarly relies on dynorphin and KOR in both CPP and self-administration paradigms (106). These results support the notion that the KOR is a pro-addictive agent during stress exposure, in contrast with its inhibitory action on acute reinforcing properties of drugs of abuse (see Part 1). As will be discussed below, clarifying this apparent paradox will require systematically determining which KOR populations are recruited in the entire brain following stress events (and following release of central CRH and systemic corticosteroids), and how this stress-induced signaling differs from KOR activation (by endogenous dynorphins or systemic pharmacological agents) in naïve, unstressed animals.

At the neuro-anatomical level, findings across several drugs of abuse and stressful modalities suggest that stress-induced reinstatement of drug-seeking relies on multiple interactions between the KOR and monoaminergic systems, as well as several forebrain limbic structures. In the DRN, results are in line with previously mentioned data on KOR-dependent CPA. Social stress-induced reinstatement of cocaine CPP was abolished after the conditional deletion of p38α in 5-HT neurons, as shown using p38α-cKO^SERT^ mice (14). In the context of nicotine addiction, FS stress-induced activation of dynorphin/KOR signaling was shown to potentiate nicotine CPP (20), an effect that could be prevented by infusion of nor-BNI in the amygdala. In the latter brain structure, recent studies have started unraveling, which neurons express the KOR (see below). Additional studies will be necessary to assess where KOR-positive amygdala neurons send projections, and whether dysregulation of nicotinic receptors, the direct nicotine targets, occurs in this or another brain region following stress exposure.

Functional interactions between NA transmission and the dynorphin/KOR system also contribute to stress-induced reinstatement of drug-seeking. Anatomical studies initially showed that the KOR is expressed in multiple cellular compartments within the LC, the main NA brain nucleus. Light and electron microscopy showed that KOR prominently co-localizes with the vesicular glutamate transporter and CRF (107), as well as with preprodynorphin (108), in axon terminals of the LC. The KOR is also expressed by LC NA neurons, as KOR immunoreactivity was found in TH-positive somatodendritic processes (108). Electrophysiological recordings indicated that KOR activation in the LC stably attenuates the neuronal activation achieved by recruiting excitatory, or CRF-positive, inputs. In contrast, KOR activation had no effect on spontaneous LC neurons activity (107), suggesting that KOR agonists predominantly recruit pre-synaptic KORs under basal conditions. At the behavioral level, KOR/NA interactions were recently investigated in the context of heroin self-administration (109). Systemic Yohimbine injection was used to precipitate relapse, based on the property of this compound to activate the HPA axis and NA neurons (acting as an antagonist at α_2_ NA inhibitory autoreceptors). Results showed that Yohimbine produced a significant reinstatement in control rats, but not in rats pre-treated with the KOR antagonist nor-BNI. Because this study used systemic administration of Yohimbine and nor-BNI, it is difficult to conclude whether the observed effects resulted from KOR blockade in the LC, or in another brain region, following Yohimbine-induced activation of the stress system (potentially leading to widespread CRF and dynorphin release). Another recent report dissected these mechanisms with better anatomical resolution, taking advantage of the simpler behavioral model of KOR agonist-induced reinstatement of cocaine CPP (39). Blockade of the KOR selectively in the LC partly prevented KOR-induced reinstatement. Consistently, rescuing the KOR in the LC of KOR KO mice partially restored KOR-dependent CPP reinstatement. Like other monoaminergic circuits (110, 111), physiological activity of NA neurons relies on multiple receptor subtypes, including inhibitory α_2_-autoreceptors and post-synaptic β_1_- and β_2_-heteroreceptors. Selective pharmacological agents were used to show that the inhibition of NA neurons (α_2_-receptor agonist), or the blockade of NA action at post-synaptic β_1_-receptors (β_1_-antagonist), both potentiated KOR-induced reinstatement. These results suggest a model whereby stress- and KOR-mediated inhibition of NA neurons contributes to relapse, and are in line with previous data showing that LC KOR activation locally decreases neuronal activity. Interestingly, both cocaine-induced reinstatement of cocaine CPP, as well as KOR-induced CPA, were unaffected by manipulations of NA signaling, suggesting that the KOR/NA interplay selectively mediates stress-related aspects of drug-seeking. In the previously mentioned study, Yohimbine precipitated relapse while it is considered an activator of both the HPA axis and NA neurons. Reconciling both studies, one might speculate that these initial stimulatory effects of Yohimbine may be followed and ultimately counteracted by CRF- and KOR-induced inhibition of LC NA neurons, leading to relapse. Finally, these data raise several questions for future studies: how are CRF-receptors and KOR interacting in the LC? Which molecular signaling pathways are recruited in LC neurons following KOR activation, and are they similar to those described in the DRN? Which forebrain structures are impacted upon LC KOR activation?

Very recently, synaptic plasticity has emerged as another level of analysis to better understand KOR-mediated reinstatement of drug-seeking. Based on previous evidence that: (i) stress impairs long-term potentiation (LTP, a form of long-lasting enhancement in synaptic transmission between two neurons) in the VTA, and that (ii) KOR regulates the mesolimbic pathway (Part 2), a recent report explored KOR modulation of LTP in the VTA as a function of stress (112). Results showed that systemic pharmacological blockade of KOR prevented stress-induced inhibition of LTP at GABAergic synapses (LTP_GABA_), but not stress-induced potentiation of excitatory synapses, within the VTA. KOR activation in the VTA was sufficient to mimic the effect of stress, and to block LTP_GABA_ in DA neurons. Importantly, intra-VTA nor-BNI infusion, *prior* to FS stress, was shown to prevent stress-induced reinstatement of cocaine self-administration. The same group of investigators further characterized the kinetics of the stress/KOR interplay (113) by looking at KOR and the glucocorticoid receptor (GR), which is activated during stressful experiences and the systemic endogenous release of corticosteroids. Following FS stress, the GR was transiently recruited (during 1 day), whereas at least 4 days of tonic activation of the KOR was necessary to mediate long-lasting effects of stress on LTP_GABA_ in DA neurons. Consistently, blocking KOR signaling *after* FS stress prevented reinstatement of cocaine self-administration. Globally, these two studies strongly suggest that GR- and KOR-dependent blockade of LTP_GABA_ in DA neurons crucially mediates stress-induced reinstatement of drug-seeking. Based on these data, it appears likely that in models of stress response and addiction, distinct plasticity processes might also occur across multiple brain regions following KOR activation.

Overall, the dynorphin/KOR system is critically implicated in relapse across a variety of animal paradigms and drugs of abuse, through complex interactions with 5-HT, DA, and NA signaling. Under baseline conditions, acute activation of the KOR inhibits the reinforcing properties of drugs of abuse (Part 1). In contrast, rodent data suggest that in humans, recruitment of the KOR during stressful life experiences may mediate reinstatement of drug-seeking in addicted individuals trying to achieve abstinence from the drug, and may therefore contribute to the maintenance of addiction.

#### Emergence of depressive symptoms in addicted individuals

Enhanced stress-reactivity during prolonged exposure to, and abstinence from, drugs of abuse contributes to the emergence of depressive symptoms, which may then evolve into chronic conditions independently from the addictive disorder.

Chronic exposure to drugs of abuse has been shown to potentiate endogenous signaling through the KOR. Repeated exposure to cocaine increased dynorphins concentrations in the striatum and substantia nigra in rats (114). Similarly, prolonged heroin self-administration led to increased *Pdyn* expression in the NAc shell and the central nucleus of the amygdala, with no effect on *Penk*, the gene encoding the enkephalin opioid peptides acting preferably at MOR and DOR (115). Chronic alcohol has also been associated with increased dynorphin expression and release in the NAc (116, 117) and the amygdala (118). As already mentioned, increased dynorphin release in the NAc likely occurs through a cAMP–CREB signaling pathway (119). Accordingly, drugs of abuse increase DA release in the NAc, leading to enhanced and prolonged activation of the DA D1-receptor, a receptor that couples to stimulatory G_s_-proteins. This in turn increases intra-cellular cAMP formation, and increases CREB binding to its genomic response elements, leading to increased expression of the *Pdyn* gene. These findings have been substantiated in humans in frontal cortical regions which, similar to the NAc, receive dense DA innervation. In a study examining post-mortem tissues from 14 alcoholics versus 14 healthy controls, increased *Pdyn* mRNA and dynorphin peptides A and B were observed in the dorsolateral PFC, as well as increased KOR mRNA in the orbito-frontal cortex, whereas, no change was found for other opioid peptides and receptors (120) in these regions.

Because of its robust prodepressant-like activity (Part 2), increased expression of the dynorphin/KOR system following prolonged exposure to drugs of abuse has been implicated in the aversive symptoms of acute withdrawal, as well as in the emergence of depressive symptoms during long withdrawal phases or abstinence. Negative affect drives drug consumption (the “self-medication” hypothesis), thereby reinforcing drug-seeking and contributing to addiction severity. In addition, drug-induced emotional disruption may also possibly lead, in vulnerable individuals, to depressive disorders evolving independently from the initial substance abuse. In rodent models, acute withdrawal from chronic ethanol exposure is associated with negative emotional states [see for examples in Ref. (121–124), including behavioral traits usually described as anxiety- (125) or depression-related (126)]. It is likely that both of these dimensions of emotional responses interact (127–129), and that withdrawal-induced anxiety-like behaviors may potentiate depressive symptomatology. In rats, ethanol-dependence can be established by chronic and passive exposure to an ethanol liquid diet (125) or to ethanol vapors (130). In Wistar rats (125), dependence has been shown to manifest as enhanced anxiety-like behavior (as assessed in the elevated plus maze test) during acute withdrawal, and this effect was blocked by systemic treatment with the KOR antagonist nor-BNI. Kissler et al. (130) also observed that acute withdrawal from ethanol-dependence associates with increased alcohol operant self-administration, and an increase in 22-kHz ultrasonic vocalizations, which represents “*an ethologically valid behavior that easily discriminates negative affective states*” (131). These behavioral changes associated with increased Dynorphin-A immunoreactivity in the capsular region of the central amygdala (CeA) and increased agonist-stimulated G-protein coupling of KOR [as measured using the classical [^35^S]-GTPγS method (132)]. Blockade of KOR in the CeA was shown to prevent escalated ethanol self-administration in dependent rats. The effect of this local manipulation on ultrasonic vocalizations was not assessed; however, it is likely that CeA KOR signaling may contribute to negative affect following chronic ethanol exposure. At the circuitry level, localization of KOR in the amygdala and its physiological relevance has only begun to be appreciated, and recent results indicate that the receptor mainly locates on pre-synaptic terminals of GABAergic neurons (133). Consistently, administration of a KOR agonist and an antagonist onto slice preparations of amygdala rat tissue, respectively decreased and increased GABAergic transmission [miniature IPSCs, (21)]. Surprisingly, these two compounds were found to have inverse effects following daily sessions of cocaine self-administration, and respectively induced increased and decreased GABAergic activity. These effects were observed only in rats that escalated cocaine consumption during long (6 h) sessions of self-administration, but not in rats showing stable cocaine consumption during short (1 h) sessions. Therefore, while chronic exposure to drugs of abuse potentiate dynorphin/KOR signaling, it is also possible that loss-of-control over drug-taking may specifically modify the net impact of KOR activation on specific neuronal circuits (as exemplified here in the CeA), possibly due to changes in cell types expressing the KOR, or in the cellular localization of KOR. At the behavioral level, CeA micro-infusion of nor-BNI attenuated the heightened anxiety-like behavior (in the defensive burying paradigm) that was observed during withdrawal from chronic, experimentally delivered, cocaine injections. While this effect of KOR blockade should also be tested following voluntary cocaine consumption, these results clearly suggest that amygdala KORs control emotional responses during cocaine withdrawal.

During the repeated cycles of intoxication and withdrawal that characterize addiction, some environmental cues progressively associate with negative affective states, and may then produce aversive effects independently of any drug exposure [even including withdrawal-like symptoms (134)]. Along this line, Berger et al. (19) showed that air-puff induced 22-kHz ultrasonic vocalizations are potentiated during withdrawal from ethanol-dependence (induced by a 2-week forced exposure to ethanol vapors), and this effect was dose-dependently blocked by systemic KOR antagonism. In another set of experiments, the authors associated a neutral odor (almond scent), with the aversive properties of systemic KOR activation. Interestingly, re-exposure to this conditioned odor was shown to potentiate ethanol operant self-administration in non-dependent rats, and this effect was blocked by KOR systemic blockade. Likewise, in humans, re-exposure to contextual cues that have been repeatedly paired with withdrawal-induced negative affect may produce a KOR-dependent dysphoric state and potentiate drug-seeking, thereby contributing to the maintenance of addiction and the emergence of depressive symptoms.

Emotional consequences of drugs of abuse extend well beyond the acute withdrawal phase, defined as the period during which the drug is cleared from the body. A recent study examined the long-term KOR-dependent changes associated with protracted withdrawal from ethanol (135). Rats were fed a liquid alcohol diet for 25–30 days, using oral self-administration in a two-bottle choice paradigm. Six weeks following ethanol removal, anxiety-like behaviors (measured immediately following a 20-min restraint stress, in the elevated plus maze) were potentiated in ethanol-abstinent rats. This effect was blocked by nor-BNI pre-treatment 24 h before testing, suggesting that increased stress-reactivity of the dynorphin/KOR system may persist for very long periods following initial ethanol exposure. Our group recently expanded this growing body of evidence to opiate abuse, and implicated KOR in emotional deficits during long-term drug abstinence in mice. We first showed that morphine abstinence progressively leads to the emergence of increased behavioral despair (in the tail suspension test) and social withdrawal (136, 137). Both deficits were detected 4 weeks, but not 1 week, following chronic experimentally delivered high morphine doses. Chronic *per os* treatment with the antidepressant Fluoxetine (a selective serotonin reuptake inhibitor) during the 4-week abstinence period reversed morphine-induced deficits. Further, 5-HT metabolism (136) and 5-HT1A-receptor function (138) were dysregulated during morphine abstinence, in particular in the DRN, suggesting an important contribution of 5-HT mechanisms. Strengthening this model, we characterized a slightly different kinetic pattern using heroin (139): at 4 weeks of abstinence, only social withdrawal was detected in heroin-pre-treated mice; at 7 weeks of abstinence, this initial symptom was accompanied by increased behavioral despair (in the FS test). Importantly, we showed that this robust decrease in social interactions (observed across both morphine and heroin abstinence) relies on the activation of both MOR and KOR (139): this phenotype was absent: (i) in cKO mice, in which the MOR was specifically deleted in the DRN prior to heroin treatment; and (ii) in constitutive KOR KO mice. Considering previous data on a 5-HT and DA interplay at the level of the NAc in models of KOR-dependent CPA and cocaine CPP, an interesting possibility is that similar monoamine interactions may contribute to emotional disruption during opiate abstinence, potentially through similar molecular cascades.

An important task for future research will be to explore emotional-like responses in the context of more sophisticated models of addictive-like behaviors. In a phylogenic and translational perspective, and using self-administration paradigms, several groups have been able to transpose DSM-IV addiction criteria into reproducible, drug-induced behavioral abnormalities, including the emergence of compulsive drug-seeking and drug-taking despite adverse consequences (140, 141). We speculate that such aberrant patterns of drug intake may also lead to stronger and more prolonged emotional deficits in rodents, and may represent better models of the emotional comorbidity associated with addiction. Such approaches also have the potential to reveal, in a dimensional approach, the behavioral traits that not only predict the transition to compulsive drug use (such as high impulsivity), but also the risk of emotional comorbidity.

Collectively, the rapidly expanding KOR literature has stimulated great interest in the development of KOR antagonists as pharmacotherapies for depression and anxiety disorders, as well as to improve stress regulation and reduce dysphoria in the context of addiction. Although some KOR ligands have not demonstrated optimal pharmacological properties, others have been shown to be viable drug candidates (142). In summary, KOR antagonists may (i) block stress-induced potentiation of drug consumption, (ii) prevent stress-induced relapse during abstinence periods, and (iii) limit negative emotional states during both acute withdrawal and more prolonged abstinence periods. Although long-term follow-ups and well-controlled studies are methodologically challenging in drug addicts, these results are coherent with a clinical report in depressed opiate abusers of the beneficial effects of buprenorphine, a dual MOR agonist/KOR antagonist (compared to methadone, a pure MOR agonist) (23); another study, however, failed to detect a difference between these two compounds (143). Intensive research in KOR pharmacology has already produced a plethora of short- [Zyklophin (103), LY-2456302 (144, 145)] and long-acting (nor-BNI, GNTI, JDTic) antagonists (146). Future studies will have to carefully analyze their respective signaling properties depending on the structural conformation they achieve with the KOR, i.e., the promising field of biased agonism [see for example in Ref. (147–150)]. Additional specificities may emerge when comparing KOR signaling across rodent and human species (151), or as a function of genetic polymorphisms (152–154). Also, the recent possibility of studying human KOR *in vivo*, using PET-Scans with the radiotracer 11C-LY2795050 (155), is promising. In the long-term, pharmacogenomic approaches have the potential to predict individualized treatment modalities targeting the KOR, and may therefore become the key to efficient clinical prescriptions.

### When depression precedes addiction

To address the neurobiological mechanisms of comorbidity between depression and addiction, another complementary approach in animal models is to study how depressive-like states may potentiate behavioral effects and patterns of consumption of drugs of abuse (36, 156). Compared to the inverse causal relationship implicated in comorbidity, the later aspect has been poorly studied, and very few studies have explored the potential role of the KOR.

In this framework, available rodent evidence is inconsistent, and chronic stress-based models of depression have been associated with either increased effects of drugs of abuse (a sensitization of reward pathways that would be consistent with the human comorbidity) or decreased effects. Krishnan et al. (93) showed that mice that are susceptible to chronic social defeat effects, who develop long-lasting depressive-like features, including decreased sucrose preference and social avoidance, also show a significant CPP at cocaine doses that are not reinforcing in undefeated mice, or in defeated but resilient mice.

Chronic mild stress is another model of depression, which is based on the unpredictable exposure of rodents to multiple mild stressors, typically over 4–8 weeks. This model is extensively used because of its face, construct, and predictive validities (157–159). The most common behavioral output in chronic mild stress (CMS) experiments is a decreased preference (over water) for a sucrose solution, or anhedonia. This anhedonic phenotype also seems to extend to the reinforcing properties of drugs of abuse, as decreased CPP for amphetamine (160) and morphine (161) has been reported following CMS in rats. Surprisingly, there is no available study, to our knowledge, on CMS effects in KOR KO mice: is KOR expression potentiated in stressed WT mice? In which brain regions? Would KOR KO mice be protected against the effects of chronic stress? Addressing this gap in the literature, Al-Hasani et al. recently explored the effects on reinstatement of CPP of three stressful modalities: CMS, a “sub-chronic social defeat” (a shorter 5-day form of social defeat), and a single acute FS stress (162). Results showed that, as previously described, acute stress potentiates KOR-mediated reinstatement of cocaine CPP. In contrast, both CMS and sub-chronic social defeat were found to attenuate KOR agonist-dependent reinstatement of cocaine and nicotine CPP. As expected, drug-induced reinstatement of cocaine or nicotine CPP was unaffected by CMS, adding to previous evidence on the specific implication of KOR in stress-induced relapse. These counterintuitive results suggest that, at least in rodent models, CMS may have protective or adaptive effects against drug relapse, a notion that fits poorly with epidemiological and clinical findings in humans.

Overall, we speculate that anhedonia-like behaviors following either CMS or prolonged social defeat may decrease the acute reinforcing properties of drugs of abuse (as assessed using place preference paradigms of drug conditioning, extinction, and relapse), possibly implicating a KOR-dependent mechanism. At the same time, stress-induced anhedonia may also potentiate the emergence of compulsive drug-taking during chronic voluntary consumption of drugs of abuse, hence favoring the entry into addiction. To explore this possibility, future studies will ideally combine two sets of advanced behavioral paradigms: CMS or chronic social defeat first, followed by extended operant drug self-administration. The plethora of cKO mice now available should prove useful in better understanding the role of KOR in these combined preclinical approaches of comorbidity.

## Future Directions and Conclusion

A major challenge in the future will be to unravel dynamic adaptations of the endogenous dynorphin/KOR system as mood and reward disruption emerge and evolve. This issue is of significant clinical relevance considering the chronicity of these two conditions. In particular, available evidence indicates that the KOR exerts multiple controls over the main monoamines in rodents. Interestingly, addiction research suggests that repeated exposure to drugs of abuse disrupts mutual inhibitory feedback mechanisms between monoaminergic nuclei, which may mediate long-term behavioral dysfunction (163, 164). Whether such mechanisms also impair KOR-dependent mood regulation is an intriguing hypothesis in the context of comorbidity.

Accumulating evidence in the KOR field has recently prompted clinicians to undertake brain imaging studies and clinical trials (22). Very recently, the first PET-Scan study using a radioactive KOR antagonist was able to demonstrate significant and widespread disruption of KOR *in vivo* availability in subjects suffering from fear and dysphoric symptoms following severe trauma exposure (165). While results are nicely consistent with animal data on KOR and the mesolimbic pathway, they also suggest that other brain regions, currently poorly explored in preclinical settings, may be equally important (e.g., thalamus and insular cortex). Additional studies will be required to further assess KOR availability in well-characterized cohorts of depressed, addicted, and comorbid subjects. Finally, from a pharmacological point of view, the rapidly evolving field of biased agonism (or ligand-directed signaling) raises great hopes for KOR-targeting therapeutics (149). A major goal in the field of G-protein-coupled receptors is the identification of distinct signaling pathways that may operate to control specific behavioral responses. In the near future, such approaches will likely aid in the development of antidepressants acting as KOR antagonists and devoid of potentially associated adverse effects (e.g., hyperalgesia).

In conclusion, we have summarized in the present review the large body of evidence supporting the role of KOR in regulating reward and mood. We have also described how this receptor is ideally placed to mediate strong interactions between two frequent and severe psychiatric disorders, addiction, and depression. Altogether, preclinical research on the KOR exemplifies how transversal studies across multiple animal models have the potential to identify brain mechanisms that contribute to transdiagnostic pathophysiological processes, and therefore represent key therapeutic targets for the management of comorbidity, one of the most prominent global issues in mental health.

## Conflict of Interest Statement

The authors declare that the research was conducted in the absence of any commercial or financial relationships that could be construed as a potential conflict of interest.
